# Is Prospective Memory Monitoring Governed by Dual Processes of Initiating a Retrieval Mode and Checking for Targets? A Conceptual Replication and Extension of Guynn (2003)

**DOI:** 10.5334/joc.508

**Published:** 2026-07-03

**Authors:** Madeline R. Valdez, Julie M. Bugg

**Affiliations:** 1 Department of Psychological and Brain Sciences, Washington University in St. Louis, US

**Keywords:** Prospective memory, Attention, Cognitive Control

## Abstract

Prospective memory (PM) is often supported by monitoring the environment for a PM target that signals it is the appropriate time to perform the intention, a process that is attentionally demanding and therefore induces a cost to the ongoing task. Consequently, monitoring is employed strategically by heightening monitoring in contexts where PM targets are likely to occur, and relaxing monitoring in other contexts. In a seminal paper, Guynn (2003) proposed a dual-process model to explain the mechanisms of strategic monitoring, positing that strategic monitoring is supported by two processes: retrieval mode and target checking. The dual-process model has since been a fundamental contributor within the PM domain, playing a critical role in both theoretical and empirical progress. Although the dual-process model has undoubtedly left its mark, Guynn’s (2003) original findings have not yet been replicated. Moreover, there has been no test of whether these results are replicable in a more traditional PM paradigm. In Experiment 1, we conceptually replicated Guynn’s (2003) findings in support of the dual-process model of strategic monitoring after adjusting her design slightly to provide a more complete characterization of the influence of strategic monitoring on ongoing task costs. Experiment 2 aimed to conceptually replicate the evidence for the dual-process model in a traditional ongoing task within the PM literature, a lexical decision task. Although we found little support for the dual-process model in lexical decision task performance, the pattern of results raises the possibility that task characteristics may affect engagement of retrieval mode and target checking. Ultimately, this study provides the first replication of Guynn (2003) and highlights the need for further investigation of the dual-process model in other PM paradigms.

## Introduction

The ability to remember to perform an intended action in response to a future event, referred to as event-based prospective memory (PM), is a challenge encountered frequently in daily life. Examples of real-life PM tasks include remembering to feed your dog when you get home from work or send an email to a colleague when you return to your office. One unique characteristic of PM is that it involves the execution of a delayed intention. That is, there is an interval between the time the intention is formed and when it must be executed. Typically, we do not have the luxury of simply waiting for the appropriate time to execute our intention, rather, this delay is often filled with other tasks. Take, for example, a student who forms an intention to turn in a homework assignment when they return to their dorm room that evening. In the meantime, they are occupied by other tasks (e.g., attending class, writing an essay, conversing with friends). Thus, successful PM requires the retrieval of the intention (i.e., to turn in a homework assignment) while actively engaged in other tasks.

To capture this characteristic of real-life PM tasks, Einstein and McDaniel (1990) developed a laboratory paradigm in which a PM task is embedded within an attentionally-demanding ongoing task, in their case, a short-term memory task. Since the inception of this paradigm, it has been adapted by many researchers to investigate the processes that support successful PM. Notably for present purposes, Smith (2003) implemented this approach, utilizing a lexical decision task (LDT) as the ongoing task, to examine the influence of PM demands on ongoing task performance. Half of the participants performed an ongoing LDT while remembering a PM intention. The other half performed the LDT only. Findings revealed that ongoing task reaction times (RT) were slower for participants who had a PM intention compared to those who did not. This slowing of ongoing task RT associated with PM performance is referred to as *cost*. Cost is thought to reflect monitoring – the deployment of resource-consuming preparatory attentional processes to monitor the environment for a PM target (i.e., cue) that signals it is the appropriate time to perform the intention (Smith, 2003).

There is a general consensus amongst theorists that monitoring is resource-demanding, and induces cost to the ongoing task (Anderson et al., 2019; Einstein & McDaniel, 2005; McDaniel et al., 2004; Scullin et al., 2013; Smith, 2003; Smith et al., 2017). Given this cost, it is generally not optimal to continuously monitor the environment for PM targets. Instead, one should monitor selectively in contexts where PM targets are likely to occur (i.e., expected PM contexts) and relax monitoring when they are not likely to occur (i.e., unexpected PM contexts). This monitoring approach, termed *strategic monitoring*, refers to the use of contextual information to guide monitoring. For example, if you intend to deliver a message to a friend, learning that you are more likely to encounter this friend on campus than at the gym allows you to selectively heighten monitoring while on campus and relax monitoring while at the gym.

Guynn’s (2003) dual-process model of strategic monitoring was developed to explain the processes that support strategic monitoring in PM. As the name suggests, this model posits that strategic monitoring is supported by two processes: *prospective memory retrieval mode* (or *retrieval mode*, for short) and *target checking*. Retrieval mode is a general readiness or predisposition to treat stimuli as cues to retrieve PM intentions. When a contextual cue signaling an expected PM context is encountered, retrieval mode is initiated and persists for all trials within the expected context. Thus, retrieval mode is maintained even if the expected context contains trials where PM targets are not likely to occur. Initiating and maintaining retrieval mode is resource-demanding and consequently induces cost to the ongoing task. The second process, target checking, involves checking relevant features of the environment (i.e., stimuli in which the target may occur) for PM targets. Although checking induces cost, it is relatively easy to initiate. Therefore, unlike retrieval mode which persists for all trials in an expected context (even those in which targets are unlikely to occur), target checking is only initiated intermittently to check for targets on relevant trials. To illustrate the dual-process model in the context of a real-life PM challenge, let’s return to the example above in which you intend to deliver a message to a friend, and have learned that you are likely to encounter this friend on campus. Therefore, you strategically monitor for this friend while on campus. For the sake of this example, let’s assume that your friend has brown hair. According to the dual-process model, retrieval mode would be initiated when you enter campus (i.e., an expected context) and would persist the entire time you are on campus, regardless of the whether the individuals you encounter have brown hair or not. However, target checking would be initiated only when you encounter an individual with brown hair (i.e., a relevant situation) and would otherwise remain inactive.

**Figure 1:**
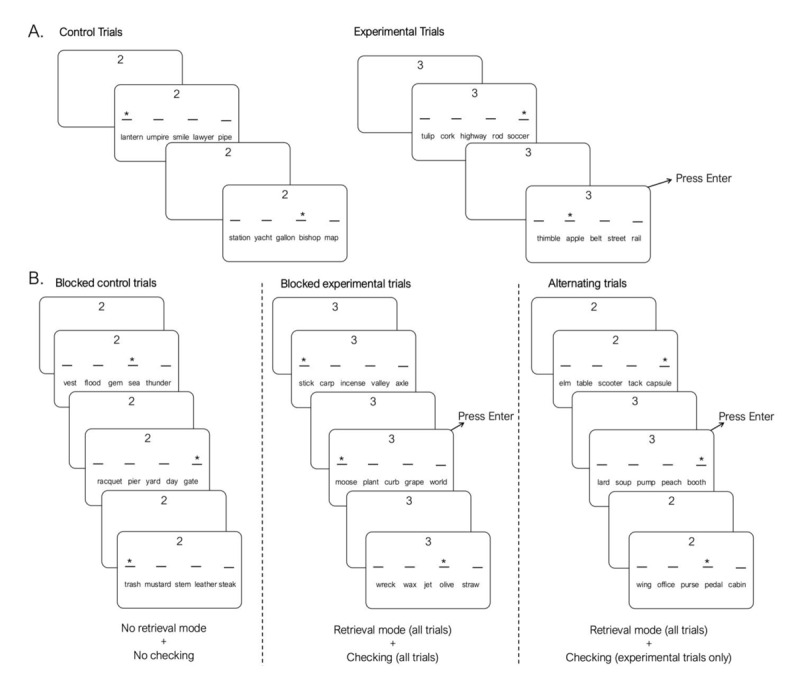
Experimental procedures from Guynn (2003). **A.** Example control and experimental trials. Participants completed the short-term memory and asterisk tasks together during control trials, which were denoted with a “2” at the top of the screen. Participants performed the short-term memory task, asterisk task, and PM task during experimental trials, which were denoted with a “3” at the top of the screen. For the PM task, participants were instructed to press Enter if a fruit word appeared. **B.** Example blocked control trials, blocked experimental trials, and alternating trials.

To test this theory, Guynn (2003) conducted one experiment in which participants performed three separate tasks: a short-term memory task, an asterisk task, and a PM task. For the short-term memory task, participants were asked to study five words on the screen and to recall as many of those words as possible once the words disappeared. For the asterisk task, an asterisk appeared in one of four locations on the screen, and participants were instructed to press a key corresponding to the location of the asterisk. The asterisk task was continuous, such that the asterisk moved to a new location every time a correct key press was made. Lastly, for the PM task, participants were told to press the *Enter* key if a fruit word appeared on the screen during the short-term memory task. Consistent with existing paradigms at the time (e.g., Einstein et al., 1992; Einstein & McDaniel, 1990), the short-term memory task acted as an ongoing “cover” task to capture the dual-task nature of PM challenges. However, this short-term memory task was not particularly well-suited to measure ongoing task reaction time, which serves as an additional footprint of monitoring. Thus, Guynn (2003) included the asterisk task to measure both ongoing task accuracy and reaction time for a more comprehensive characterization of monitoring. Lastly, the PM task was implemented to measure cost to ongoing task performance in response to PM demands, in addition to measuring PM performance. During the experiment, participants completed two different types of trials: control and experimental (see Figure 1A for examples of each trial type). On control trials, participants performed the ongoing tasks together (i.e., the short-term memory and asterisk tasks). A “2” appeared at the top of the screen on control trials to signify that participants should perform both tasks. On experimental trials, participants performed the PM task in addition to the ongoing tasks. A “3” appeared at the top of the screen during experimental trials to indicate that participants should perform all three tasks. Critically, the experiment was designed such that control and experimental trials were either blocked (i.e., consecutive trials of the same type) or alternating (i.e., alternating between control and experimental trials on every other trial). Figure 1B shows example blocked control trials, blocked experimental trials, and alternating control and experimental trials.

For blocked control trials (see leftmost panel of Figure 1B for example), participants completed 24 consecutive control trials. According to the dual-process model, participants should not be in retrieval mode nor check for targets during blocked control trials. During blocked experimental trials (see middle panel of Figure 1B for example), participants completed 24 consecutive experimental trials. According to the model, participants should be in retrieval mode and check for targets during blocked experimental trials. During alternating trials (see rightmost panel of Figure 1B for example), participants completed 48 control and experimental trials that alternated every trial. Here the model posits that participants should maintain retrieval mode during all alternating trials, regardless of whether they are control or experimental, but should check for targets only during experimental trials.

**Figure 2:**
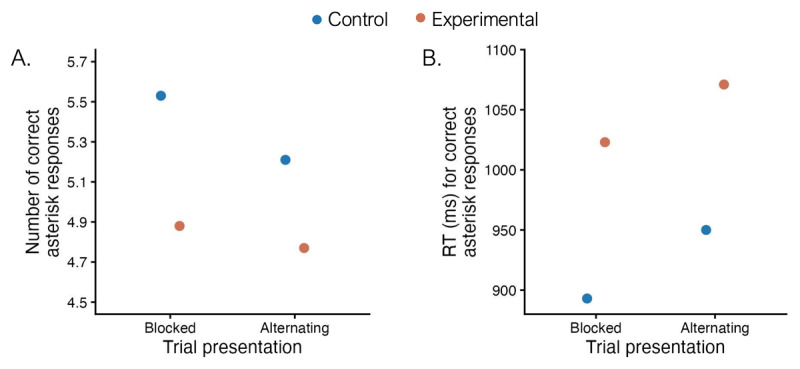
Asterisk task performance results from Guynn (2003). **A.** Average number of correct asterisk task responses per run during blocked and alternating control and experimental trials. **B.** Average reaction time on asterisk task during blocked and alternating control and experimental trials. Figure recreated from Guynn (2003).

Figure 2A depicts asterisk task accuracy results from Guynn (2003). When discussing the results below, we include the *F* statistics reported by Guynn (2003) for each of the primary effects from a 2 (trial type: control, experimental) × 2 (trial presentation: blocked, alternating) repeated measures ANOVA, as well as their associated *p*-value and effect size (partial eta squared; η_p_^2^).^1^ There was a main effect of trial type driven by lower asterisk task accuracy during experimental trials relative to control trials, *F*(1, 63) = 97.55, *p* < .001, *MSE* = 0.20, η_p_^2^ = .61, signifying a cost to this ongoing task during trials where participants were expected to monitor for PM targets. There was also a main effect of trial presentation, *F*(1,63) = 13.34, *p* < .001, *MSE* = .11, η_p_^2^ = .17, driven by lower asterisk task accuracy during alternating relative to blocked trials. Additionally, and most critically, there was an interaction between trial type (i.e., control, experimental) and trial presentation (i.e., blocked, alternating), *F*(1, 63) = 6.32, *p* = .015, *MSE* = 0.11, η_p_^2^ = .09. This interaction was decomposed. For control trials, it was found that asterisk task accuracy was lower on alternating trials compared to blocked trials, *F*(1, 63) = 7.45, *p* = .008, *MSE* = 0.11, η_p_^2^ = .11. Thus, despite not needing to allocate cognitive resources to the PM task during control trials, there was a cost to ongoing task accuracy when these trials were intermixed with experimental trials. This pattern supports the dual-process model, which predicts that participants should not be in retrieval mode nor check for targets during blocked control trials, evidenced by the least impairment during these trials. However, during alternating control trials, participants should be in retrieval mode because this state persists for the duration of alternating trials, though they would not be expected to check for targets. In contrast, for experimental trials, there was not a difference between alternating and blocked trials, *F*(1, 63) = .88, *p* = .648, *MSE* = 0.11, η_p_^2^ = .01. This pattern is also consistent with the dual-process model because participants should be in retrieval mode and checking for targets during all experimental trials, regardless of trial presentation. Thus, performance on experimental trials is similar for the alternating and blocked presentation.

Guynn’s (2003) dual-process model of strategic monitoring extended existing theoretical perspectives by conceptualizing the existence of a monitoring system that adapts strategically to contextual cues to support PM, identifying the two mechanisms that support it and providing initial evidence for the operation of these mechanisms. Since its instantiation, the dual process model has been a major contributor to empirical and theoretical progress within PM research (314 reported citations on Google Scholar as of May 2026). Many researchers have since used this model to motivate research questions or explain findings (Ball et al., 2020, 2024; Ball & Bugg, 2018a, 2018b; Bowden et al., 2021; Bugg & Ball, 2017; Lourenço et al., 2013; Marsh et al., 2006; Peper & Hunter Ball, 2023; Smith & Bayen, 2006). Although its influence is indisputable, there are several limitations to Guynn’s (2003) formative study that we aimed to address in two experiments in the proposed study.

The first limitation regards the fact that the cost predictions from the dual-process model were evident in cost to ongoing task accuracy, but not ongoing task RT, although the RT cost patterns were numerically consistent (Figure 2B). This is surprising given that monitoring cost is typically reflected in impairment of ongoing task RT, but not accuracy (Anderson et al., 2019; Bugg & Ball, 2017; Einstein & McDaniel, 2005; Kuhlmann & Rummel, 2014; Lourenço et al., 2013; Lourenço & Maylor, 2014; Marsh et al., 2006). One possible explanation for this finding is the way in which asterisk task accuracy and reaction time were measured. During the asterisk task, participants had 9.5 seconds (the duration of one short-term memory trial) to respond to as many asterisks as possible. Importantly, only correct responses (i.e., trials where participants successfully identified the location of the asterisk) were recorded. Therefore, the only way to measure accuracy was by the total number of correct responses, as opposed to the proportion of correct responses. Guynn (2003) acknowledges that, because of this design, both the RT and accuracy measures capture aspects of response latency and response correctness. For example, making many incorrect responses will inherently slow RTs, and having fast RTs will allow for more opportunities to make a correct response. Because there is no way to dissociate between the actual influence of PM demands on accuracy and RT, the true effects remain unclear. Therefore, one goal of the current study was to replicate the findings from Guynn (2003) while using independent measures of ongoing task RT and accuracy.

Another limitation concerns the fact that cost was only measured using performance on the asterisk task. As described above, participants in Guynn (2003) completed two ongoing tasks (i.e., a short-term memory and an asterisk task). Therefore, impairment to ongoing task performance could be measured in terms of cost to asterisk task performance, and cost to short-term memory performance. However, Guynn (2003) did not report or analyze cost to short-term memory performance, focusing instead on cost to asterisk task performance. Consequently, the influence of PM demands on ongoing task performance may not be entirely represented by the findings. It is possible (and perhaps likely) that cost to short-term memory task performance is consistent with cost to asterisk task performance, further supporting a dual-process model of strategic monitoring. However, we cannot rule out the possibility that short-term memory cost contradicted the reported cost effects for the asterisk task, which warrants further investigation.

To address these concerns, Experiment 1 aimed to replicate Guynn’s (2003) seminal findings in support of a dual-process model of strategic monitoring while providing a more comprehensive characterization of the influence of strategic monitoring on ongoing task performance. In Experiment 2, we aimed to test whether the key findings can be replicated using a single, traditional ongoing task, namely an LDT. Many studies investigating strategic monitoring processes substantiate their findings under the dual-process model view. Importantly, most of these studies use LDTs as the ongoing task (Ball & Bugg, 2018a, 2018b; Bugg & Ball, 2017; Cohen et al., 2012; Lourenço et al., 2013; Marsh et al., 2006; Peper & Hunter Ball, 2023), and although they replicate a subset of the cost patterns predicted by a dual-process model, none of them include the full set of conditions used in Guynn’s original design (i.e., blocked control and experimental trials, and alternating control and experimental trials), which is critical for testing the existence of both retrieval mode and target checking processes. For example, some studies include the equivalent of alternating control and experimental trials, but not blocked control and experimental trials (Ball & Bugg, 2018a, 2018b; Cohen et al., 2012; Lourenço et al., 2013). According to the dual-process model, differences in cost during alternating control and experimental trials reflect the target checking process. Thus, these studies (Ball & Bugg, 2018a, 2018b; Cohen et al., 2012; Lourenço et al., 2013) can test for the existence of a one-process model in which strategic monitoring is supported by target checking. However, because the designs do not include blocked control and experimental trials, they are unable to assess whether retrieval mode is also a necessary process for strategic monitoring, and therefore are limited in their ability to test the dual-process model. On the contrary, other studies include the equivalent of blocked control and experimental trials, but not alternating control and experimental trials (Bugg & Ball, 2017; Marsh et al., 2006). According to the dual-process model, the difference in cost between blocked control and experimental trials reflects both retrieval mode and target checking processes. However, this conclusion can only be assessed by comparing differences in cost between blocked *and* alternating experimental and control trials, as it is the interaction between these conditions that is a true test of a dual-process model. So, while it’s possible the results from Bugg and Ball (2017) and Marsh et al. (2006) do indeed reflect a dual-process model, they could instead reflect a one-process model in which strategic monitoring is driven by retrieval mode only or target checking only. Consequently, it remains unclear whether the cost patterns revealed in these studies reflect both processes of the dual-process model. Therefore, it is critical to determine if evidence for a dual-process model is replicable in the context of PM paradigms that utilize the full set of conditions necessary to test for the presence of a dual-process model.

## Experiment 1

The main goal of Experiment 1 was to replicate the findings from Guynn (2003) supporting a dual-process model of strategic monitoring. For the large part we preserved the original paradigm used by Guynn, but attempted a conceptual replication rather than a direct replication because we made several slight adjustments to provide a more comprehensive characterization of the costs associated with strategic monitoring, and thereby a more comprehensive test of the theory. First, we adjusted the procedure of the asterisk task so that incorrect responses were recorded. Therefore, accuracy could be measured as the proportion of correct responses, and reaction time was a purer reflection of response latency. This allowed us to analyze ongoing task accuracy and reaction time independently. Second, we analyzed not only costs on the asterisk task but additionally costs on the short-term memory task. This allowed us to examine whether patterns of monitoring, when considering both tasks, remain consistent with the predictions from the dual-process model.

### Predictions

As described earlier in the Introduction, Guynn (2003) found that the cost pattern predicted by the dual-process model was reflected in asterisk task accuracy, but not RT (although it was numerically present). This contradicts findings from existing research on monitoring cost showing the opposite: PM demands often induce cost to ongoing task RT but not accuracy (e.g., Anderson et al., 2019; Bugg & Ball, 2017; Einstein & McDaniel, 2005; Kuhlmann & Rummel, 2014; Lourenço et al., 2013; Lourenço & Maylor, 2014; Peper & Hunter Ball, 2023). One potential explanation for Guynn’s (2003) findings is the fact that asterisk task accuracy and RT measures reflected aspects of response latency as well as correctness. In Experiment 1, we adapted the original design such that response latency and correctness are independent measures. If the results of Experiment 1 replicate Guynn (2003), we should find that the cost pattern is reflected in asterisk task accuracy. While this would be inconsistent with the existing research referenced above, it would still provide evidence of a dual-process account of strategic monitoring. Indeed, a large portion of the literature showing cost to ongoing task RT and not accuracy utilizes LDT as the ongoing task while other work utilizing alternative ongoing tasks, such as color matching tasks (e.g., Smith & Bayen, 2006), shows cost to ongoing task accuracy. Therefore, Guynn’s (2003) findings of cost to ongoing task accuracy but not RT may instead reflect properties of the ongoing task (e.g., speed-accuracy tradeoffs associated with a given task). For example, participants may have prioritized responding consistently fast across all trials at the expense of responding accurately. That is, during trials in which participants were not in retrieval mode nor target checking (i.e., blocked control trials), they could respond quickly *and* accurately because all attentional resources were allocated to the ongoing task. In contrast, during trials in which participants were in retrieval mode (i.e., alternating control trials) or in retrieval mode and target checking (i.e., experimental trials), they may have prioritized responding quickly, but consequently made more errors because they had to divide attentional resources between the ongoing task and monitoring for PM targets. In this case, the expected cost pattern would be reflected in accuracy, but not RT, as Guynn found.

However, given prior research (Anderson et al., 2019; Bugg, 2017; Einstein & McDaniel, 2005; Kuhlmann & Rummel, 2014; Lourenço et al., 2013; Lourenço & Maylor, 2014; Peper & Hunter Ball, 2023) and the modified procedure in Experiment 1, we might alternatively expect that the cost pattern would be reflected in asterisk task RT. Although a cost pattern reflected in ongoing task RT but not accuracy would not directly replicate the findings from Guynn (2003), it would align with predictions derived from the dual-process model. Additionally, it would help explain the discrepancy between accuracy and RT cost patterns demonstrated in Guynn (2003) and those demonstrated in other research (Anderson et al., 2019; Bugg & Ball, 2017; Einstein & McDaniel, 2005; Kuhlmann & Rummel, 2014; Lourenço et al., 2013; Lourenço & Maylor, 2014). To convey the possibility that cost could be reflected in either (or both) ongoing task accuracy and RT, we describe the predicted cost patterns as cost to ongoing task “performance”, rather than specifying cost to accuracy or RT. Specifically, the dual-process model predicts that participants should maintain a retrieval mode and check for PM targets during blocked and alternating experimental trials, as these are contexts in which PM targets are expected to occur. Therefore, we expected a decrement to asterisk task performance during experimental trials relative to control trials (i.e., a main effect of trial type). Additionally, the dual-process model predicts that participants should maintain retrieval mode but not check for targets during alternating control trials. Thus, we expected a decrement to asterisk task performance during alternating control trials, where participants are expected to be in retrieval mode, relative to blocked control trials, where participants are not expected to be in retrieval mode. Critically, this trial presentation difference is not expected to extend to experimental trials, as participants should be in retrieval mode and target checking during all experimental trials. Thus, asterisk task performance was expected to be equivalent in blocked and alternating experimental trials. Together, these predictions were expected to result in an interaction between trial type and trial presentation, driven by a larger difference between blocked and alternating control trials relative to the difference between blocked and alternating experimental trials. It should also be noted that Guynn (2003) reported a significant main effect of trial presentation (a decrement to performance for the alternating compared to blocked presentation). Although not a theoretically important effect on its own, it is nonetheless expected given Guynn’s (2003) results.

We also considered the possibility that our results would not replicate a dual-process model and may instead be consistent with a one-process model under which strategic monitoring is guided by retrieval mode *or* target checking, but not both. If strategic monitoring is solely driven by retrieval mode, then we expected participants to be in retrieval mode during alternating control trials and all experimental trials, but not during blocked control trials. In this case, there would be an equal decrement to asterisk task performance during all experimental trials and alternating control trials relative to blocked control trials. An alternative one-process model would be one in which strategic monitoring could be supported by target checking only. This model would predict that participants check for targets on experimental trials but not on control trials, and therefore asterisk task performance during experimental trials would be worse compared to control trials, regardless of whether the trials are alternating or blocked. Evidence favoring either of these one-process models would have important theoretical implications, suggesting that successful PM monitoring does not necessitate the initiation of both retrieval mode and target checking, as either process alone could be sufficient for supporting PM monitoring.

### Method

The materials and procedures used in this study were nearly identical to those in Guynn (2003), with several exceptions. To briefly summarize the deviations from the original paradigm, PM targets during the practice phase were changed from focal to nonfocal targets, participants were given feedback on their performance during the practice phase for the asterisk task and PM task, short-term memory accuracy was recorded and analyzed, and the asterisk task was modified so that all responses (whether correct or incorrect) were recorded. Each deviation is explained in more detail in the relevant sections below.

#### Participants and Design

An a priori power analysis was conducted (Gpower, v.3.1; Franz et al., 2007) to determine the sample size needed to detect a significant interaction between trial type (experimental vs. control) and trial presentation (blocked vs alternating) using a two-way repeated measures ANOVA. Based on the findings from Guynn (2003), the size of this interaction effect on ongoing task accuracy is moderate (η_p_^2^ = .09). Because we examined this interaction for ongoing task accuracy and RT (for which the effect size is unknown), we decided to use a more conservative effect size (η_p_^2^ = .07). To detect an effect of this magnitude with 90% power and an alpha of .05, we needed a total of 136 participants. To achieve this, 136 participants were recruited from Washington University’s psychology subject pool to complete this experiment for course credit. All participants completed informed consent prior to the start of the experiment and all materials and procedures were approved by Washington University’s Institutional Review Board. Participant demographics are reported in Table 1.

**Table 1:** Participant Demographic Information.


	EXPERIMENT 1	EXPERIMENT 2

N	136	136

Age *M* (*SD*)	19.8 (1.3)	19.0 (1.2)

Gender *n* (%)		

Female	97 (71%)	104 (76%)

Male	39 (29%)	32 (24%)

Race *n* (%)		

White	54 (40%)	60 (44%)

Asian	46 (34%)	47 (35%)

Multi-racial	16 (12%)	6 (4%)

Black or African American	11 (8%)	11 (8%)

Hispanic or Latino	6 (4%)	7 (5%)

American Indian or Alaska Native	0 (0%)	1 (1%)

Prefer not to answer	3 (2%)	4 (3%)


*Note*. Age is reported in years.

This was a 2 (trial type: control, experimental) × 2 (trial presentation: blocked, alternating) repeated-measures design. During experimental trials, participants completed three separate tasks: a short-term memory task, an asterisk task, and a prospective memory task. During control trials, participants only completed the short-term memory and asterisk tasks. Experimental and control trials were presented as either blocked (i.e., consecutive trials of the same type; see Figure 1B, leftmost and middle panels) or alternating (i.e., alternating between trial types; see Figure 1B, rightmost panel). The order of trial type (experimental or control) and trial presentation (blocked or alternating) was counterbalanced across participants to form four conditions: (1) blocked control, blocked experimental, alternating, (2) blocked experimental, blocked control, alternating, (3) alternating, blocked control, blocked experimental, and (4) alternating, blocked experimental, blocked control.

#### Materials

Word stimuli for the short-term memory task were the same words used in Guynn (2003), which consisted of 540 one- and two- syllable nouns from Clusters 6, 7, and 8 of Toglia and Battig (1978) semantic word norms. For each participant, 60 words were randomly selected for practice trials, and the remaining 480 words were used for test trials. For each short-term memory trial, five words were randomly chosen (without replacement) with the requirement that there were at least two one-syllable words on every trial. PM targets were eight unique fruit words (apple, peach, banana, plum, cherry, grape, apricot, pear) and appeared on 8 of the 48 experimental trials. A separate set of PM targets (animal words: dog, lion, bear, cat) was used to practice the prospective memory task during the practice phase.

#### Procedure

All experimental tasks were completed by the participants on a computer in an individual testing room. An experimenter was present in the testing room for the entirety of the experiment to answer questions regarding task instructions and to record responses to the short-term memory task.

Prior to the beginning of the study, participants were informed that they were going to complete several different tasks concurrently, and would practice performing each task separately, and then together. Participants first practiced the asterisk task. They were instructed that an asterisk would appear on the screen in one of four positions, and that they should press a button indicating where on the screen it appeared as quickly and accurately as possible. Participants received feedback on the speed and accuracy of their response after each key press during asterisk task practice. Participants were instructed to use the index and middle fingers of each hand to press the *Z* key if the asterisk appears in the first position (leftmost), the *X* key if the asterisk appears in the second position (second from the left), the *N* key if the asterisk appears in the third position (second from the right), and the *M* key if the asterisk appears in the fourth position (rightmost). The asterisk remained on the screen until the participant made a key press, after which the asterisk moved to one of the other three locations (i.e., location could not be repeated on consecutive trials), randomly chosen on each trial. This deviates from Guynn’s (2003) original design. In Guynn (2003), the asterisk remained on the screen until a *correct* response was made, whereas, in the current experiment, the asterisk remained on the screen until any response (correct or incorrect) was given. Participants received feedback on the accuracy and speed of their response during the asterisk task, which differs from Guynn’s (2003) design. However, it is important that participants received corrective feedback to help ensure they were performing the task properly before beginning the actual test trials. Each practice run lasted 9.5 seconds (i.e., the duration of a short-term memory task trial), during which asterisks continued to appear after each key press and feedback, as described above. Participants completed six practice asterisk task runs.

Participants then practiced the short-term memory task. They were instructed that five words would be presented in a row in the center of the screen for 5 seconds and that they should study the words while they are on the screen. Participants were instructed that, once the words disappear from the screen, they would have 4.5 seconds to recite, in order, as many of the words as possible. The participants’ responses (which words were recalled and in what order) were recorded by an experimenter sitting in the room. Participants completed 6 short-term memory trials, each lasting a total of 9.5 seconds, with 5 seconds to study the words and 4.5 seconds to recall the words. There was a 1 second interval between trials.

Next, participants received practice completing the asterisk and short-term memory tasks together (i.e., control trials). Participants were instructed that they should press the key corresponding to the location of the asterisk while studying and reciting the words. Participants no longer received feedback on their asterisk task performance. Importantly, participants were told that a “2” appearing at the top of the screen before the start of a trial would signal that they should perform both the asterisk and short-term memory tasks together. The “2” appeared 1.5 seconds prior to the start of each trial and remained on the screen for the duration of the trial. Participants completed 2 identical practice blocks of 6 runs, with a 10 second break between blocks.

To conclude the practice portion of the experiment, participants were informed that they would sometimes be asked to complete a third task (i.e., the PM task) as they performed the asterisk and short-term memory tasks (i.e., experimental trials). They were instructed that they should press the *Enter* key if an animal word appeared on the screen during the short-term memory task. Target words were *dog* and *lion* for the first practice block, and *bear* and *cat* for the second practice block. Participants were instructed that a “3” appearing at the top of the screen before the start of a trial would signal that they should perform all three tasks simultaneously. The “3” appeared 1.5 seconds before the start of the trial and remained there for the duration of the short-term memory trial. Participants completed two identical blocks of six practice runs. If a participant failed to detect a target word during these practice trials, they received feedback reminding them of the PM task.

Notably, the practice PM target words used in the current study differed from those in Guynn (2003). During practice experimental trials, participants in Guynn (2003) were told to press *Enter* if they saw *rake, truck, nose*, or *soap*. These targets are considered *focal* because the ongoing task requires the processing of target features that were processed during initial encoding. Because participants processed the features of the target words during encoding, and the short-term memory task requires processing of word features, these targets are focal. The target words used in test experimental trials (i.e., fruit words), however, are *nonfocal* targets. Nonfocal targets are not processed during initial encoding. That is, participants are told to look for fruit words during PM encoding, but the specific target words are not given and thus not processed. The distinction between focal and nonfocal targets is important, as previous research shows that focal target detection is supported by spontaneous retrieval, while non-focal target detection requires engagement of monitoring (Einstein & McDaniel, 2005; Scullin et al., 2013). Given the focus of this study is on strategic monitoring, we thought it was important that practice experimental trials utilized nonfocal targets to mimic experimental trials during test.

Following practice, participants were informed that they would complete test trials, which would be organized into blocks of 6 short-term memory trials, and they would receive a 10-second rest between blocks. They were instructed that, as previously practiced, some trials would require them to perform the asterisk and short-term memory tasks together (i.e., control trials). Participants were told that they would not receive feedback on the asterisk task during test trials. They were reminded that these trials would be marked with a “2” at the top of the screen. Other trials would require them to perform the asterisk, short-term memory, and prospective memory tasks together (i.e., experimental trials). These trials were marked with a “3” at the top of the screen. During test trials, for the prospective memory task, participants were instructed to press the *Enter* key if they saw a word that is a fruit. Participants were reminded of these instructions before blocked experimental trials and before alternating trials. Additionally, before the start of blocked and alternating blocks, participants were informed about whether the upcoming block would be blocked or alternating.

The test phase consisted of 16 blocks of 6 short-term memory test trials, for a total of 96 trials. The order of trial presentation (blocked vs. alternating) was counterbalanced across participants as follows. For half the participants, the first 48 trials consisted of 24 blocked control trials and 24 blocked experimental trials, followed by 48 alternating control and experimental trials. For the other half of the participants, the first 48 trials consisted of alternating control and experimental trials, followed by 24 blocked control trials and 24 blocked experimental trials. Four of the eight PM targets appeared during the blocked experimental trials, and the other four PM targets occurred in the alternating experimental trials. Moreover, the order of trial type (control vs. experimental) was counterbalanced such that, of the participants who did blocked trials first, half started with blocked control trials and the other half started with blocked experimental trials. Similarly, half of the participants who started with alternating trials began with an experimental trial and the other half began with a control trial. The first trial of alternating and blocked trials was the same. That is, if a participant started with a blocked experimental trial, they also began with an alternating experimental trial, and vice versa for those who began with a control trial.

#### Statistical Analyses

All statistical analyses were conducted in R Statistical Software (v. 4.4.2, R Core Team, 2023). Following Guynn (2003), asterisk task trials with an RT less than 100 ms were excluded from all analyses. Additionally, PM target trials were excluded from analysis of the asterisk task and short-term memory task to account for any bias that occurs as a result of PM target presentation. For all analyses, we report effect sizes for t-tests (Cohen’s d) and ANOVAS (partial eta squared; η_p_^2^). All hypothesis testing was done using an alpha of 0.05. For reaction time analyses, incorrect trials were excluded from the analysis.

##### Asterisk Task Performance

###### Accuracy

Our primary measure of asterisk task accuracy was the proportion of correct responses during each trial. However, following Guynn (2003), we also measured accuracy as the total number of correct responses to test whether Guynn’s (2003) original measure of accuracy replicates. We submitted both asterisk task accuracy measures (i.e., proportion of correct responses and total number of correct responses) to separate 2 (trial type: control, experimental) × 2 (trial presentation: blocked, alternating) repeated-measures ANOVAs. If cost is reflected in asterisk task accuracy, we expected our results to align with Guynn (2003), showing a significant main effect of trial type (accuracy during experimental trials < accuracy during control trials) and trial presentation (accuracy during alternating < accuracy during blocked). These main effects would evidence that there is a cost to monitoring, however, they cannot specify the source of this cost (i.e., retrieval mode, target checking, or both). Rather, it is a significant interaction between trial type and trial presentation that can dissociate between retrieval mode and target checking processes. If strategic monitoring is indeed supported by dual processes, then we expected there to be an interaction between trial type and trial presentation, and that this interaction would be driven by a larger difference in asterisk task performance between blocked and alternating control trials than between blocked and alternating experimental trials. That is, when decomposing this interaction, we expected a significant difference in performance between alternating and blocked control trials (accuracy during alternating < accuracy during blocked), but not between alternating and blocked experimental trials.

###### Reaction Time

Reaction time for correct asterisk task trials was compared using a 2 (trial type: control, experimental) × 2 (trial presentation: blocked, alternating) repeated-measures ANOVA. If there is a cost to asterisk task RT, we expected the patterns to be consistent with those outlined for ongoing task accuracy. Specifically, we expected main effects of trial type and trial presentation, showing that monitoring induces a cost to the asterisk task RT. More importantly, we expected an interaction between trial type and trial presentation, following the same pattern described above for accuracy when decomposed, which would evidence the existence of a dual-process model of strategic monitoring.

##### Short-term Memory Accuracy

Short-term memory accuracy was measured as the number of correctly recalled words on each trial. We compared short-term memory accuracy using a 2 (trial type: control, experimental) × 2 (trial presentation: blocked, alternating) repeated-measures ANOVA to test whether short-term memory accuracy varied depending on PM demands or whether the trials were blocked or alternating. Note that this deviated from the methods originally used in Guynn (2003) who did not report short-term memory accuracy. However, we believed that analyzing short-term memory accuracy was necessary in that it provided another footprint of monitoring cost. If monitoring cost impairs short-term memory performance, we expected results from this ANOVA to align with those from the asterisk task performance, showing significant main effects of trial type and trial presentation as well as a significant interaction between the two that follows the pattern described above.

##### Prospective Memory Accuracy

We measured PM accuracy as the proportion of successfully detected PM targets during experimental trials. We compared PM accuracy during blocked and alternating experimental trials using a paired-samples t-test to determine if PM accuracy varied depending on whether the trial alternated with control trials or was blocked. Consistent with findings from Guynn (2003), we did not expect PM accuracy to differ depending on whether the experimental trials were blocked or alternating.

### Results

The registered hypotheses, methods, and analyses, as well as the data and analysis code are publicly available on OSF using the following link: https://osf.io/9vrzu/.

#### Asterisk Task Performance

Excluding trials with an RT < 100 ms resulted in the removal of roughly .1% of asterisk task trials from accuracy and RT analyses.

##### Accuracy

**Figure 3:**
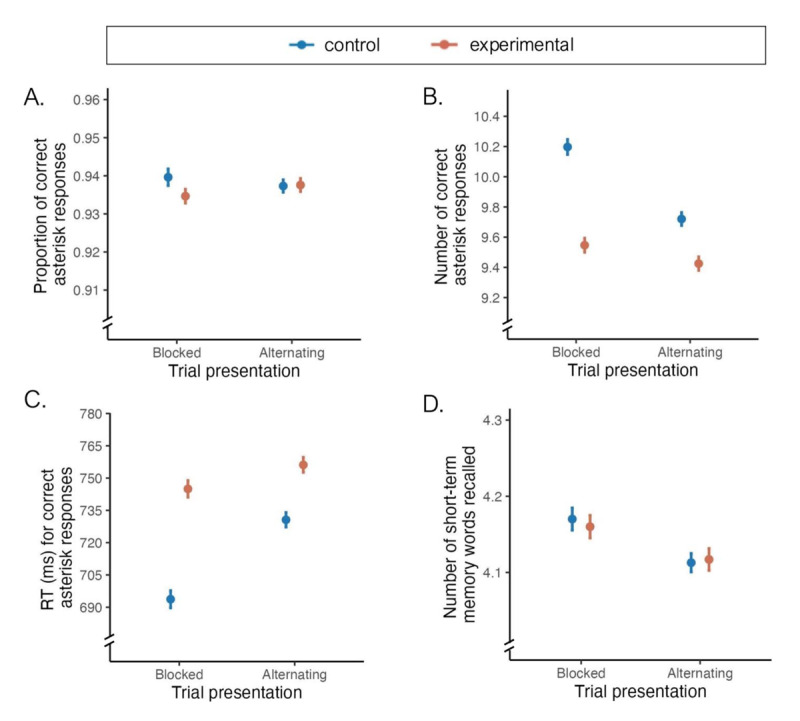
Ongoing asterisk and short-term memory task performance during Experiment 1. **A.** The mean proportion of correct asterisk presses. Larger proportions indicate better performance. **B.** The average number of correct asterisk responses. Means were calculated based on the number of correct asterisk responses made during a single short-term memory trial, and larger numbers indicate better performance. **C.** Mean reaction time for correct asterisk responses. Lower numbers indicate faster responding and thus better performance. **D.** Average number of words recalled on each short-term memory trial. All error bars represent the within-participant standard error.

We first assessed asterisk task accuracy as the proportion of correct asterisk task responses for each trial type and trial presentation, which is reported in Table 2 and depicted in Figure 3A. We submitted the proportion of correct asterisk task trials to a 2 (trial type) × 2 (trial presentation) repeated-measures ANOVA, and report the full results in Table 3. Both main effects and their interaction were not significant, indicating that evidence of a dual process model was not reflected in proportion of correct asterisk task responses.

**Table 2:** Mean Performance (Standard Deviation) Across Trial Conditions in Experiment 1 and 2.


EXPERIMENT	DEPENDENT VARIABLE	BLOCKED		ALTERNATING	
	
		CONTROL	EXPERIMENTAL	CONTROL	EXPERIMENTAL

1	Asterisk task, proportion correct	.94 (.06)	.94 (.06)	.94 (.06)	.94 (.06)

	Asterisk task, number correct	10.2 (2.3)	9.6 (2.3)	9.7 (2.2)	9.4 (2.2)

	Asterisk task, reaction time	694 (153)	744 (188)	731 (186)	756 (188)

	Short-term memory task, number correct	4.2 (.59)	4.2 (.63)	4.1 (.61)	4.1 (.63)

	Prospective memory accuracy	—	.84 (.23)	—	.78 (.28)

2	LDT, proportion correct	.94 (.05)	.93 (.05)	.94 (.05)	.93 (.05)

	LDT, reaction time	625 (101)	678 (102)	692 (133)	696 (123)

	Prospective memory accuracy	—	.61 (.31)	—	.60 (.32)


*Note*. Asterisk task reaction time is reported in milliseconds. The number correct on the short-term memory task reflects the number of correctly recalled words per trial, with a maximum possible score of five (i.e., the number of words presented on each trial). Prospective memory accuracy is not reported for control trials, as no PM targets appeared on these trials.

**Table 3:** Analyses of Variance Results from Experiment 1 and 2.


EXPERIMENT	DEPENDENT VARIABLE	EFFECT	*F*	df	*P*	η*_P_^2^*

1	Asterisk task, proportion correct	Trial type	1.05	1,135	.308	.01

		Trial presentation	.20	1,135	.653	<.01

		Trial type × Trial presentation	2.11	1,135	.149	.02

	Asterisk task, number correct	Trial type*	92.34	1,135	<.001	.41

		Trial presentation*	19.31	1,135	<.001	.13

		Trial type × Trial presentation*	19.32	1,135	<.001	.13

	Asterisk task, reaction time	Trial type*	92.65	1,135	<.001	.41

		Trial presentation*	21.25	1,135	<.001	.14

		Trial type × Trial presentation*	13.33	1,135	<.001	.09

	Short term memory task, number correct	Trial type	.06	1,135	.860	<.01

		Trial presentation*	8.34	1,135	.005	.06

		Trial type × Trial presentation	.23	1,135	.636	<.01

2	LDT, proportion correct	Trial type*	13.39	1,135	<.001	.09

		Trial presentation	.88	1,135	.351	.01

		Trial type × Trial presentation	.01	1,135	.754	<.01

	LDT, reaction time	Trial type*	90.12	1,135	<.001	.40

		Trial presentation*	27.52	1,135	<.001	.17

		Trial type × Trial presentation*	44.59	1,135	<.001	.25


*Note*. All analyses of variance were 2 (trial type) × 2 (trial presentation) repeated-measures ANOVAs. * denotes a significant effect.

Next, we examined the average number of correct asterisk responses, which is reported in Table 2 and depicted in Figure 3B separately for each trial type and trial presentation. We submitted the number of correct asterisk responses to a 2 (trial type) × 2 (trial presentation) repeated-measures ANOVA and report the full results in Table 3. There was a main effect of trial type, driven by a larger number of correct asterisk responses during control trials relative to experimental trials. The main effect of trial presentation was also significant and was driven by a larger number of correct responses during blocked compared to alternating trials. Finally, the interaction between trial type and trial presentation was significant. Preregistered post-hoc comparisons revealed a significant difference in the number of correct responses during blocked and alternating control trials (blocked control > alternating control, *M_diff_* = .17), *t*(135) = 5.94, *p* < .001, *d* = .51, but not during blocked and alternating experimental trials (*M_diff_* = .12), *t*(135) = 1.56, *p* = .122, *d* = .13. We also examined whether the number of correct responses differed between alternating control and experimental trials (i.e., the simple effect of trial type within alternating trials). Although this comparison was not preregistered because it was not examined in Guynn (2003), we thought it was important to test because a key prediction of the dual process model is that performance should be better on alternating control trials, where participants are in retrieval mode but not target checking, relative to alternating experimental trials, where participants are in retrieval mode and target checking. Consistent with this prediction, a paired-sample t-test revealed that participants made more correct responses during alternating control trials compared to alternating experimental trials, *t*(135) = 5.19, *p* < .001, *d* = .44. Collectively, these patterns demonstrate evidence of a dual-process model in the number of correct asterisk task responses.

##### Reaction Time

Mean asterisk task RTs for each trial type and trial presentation are reported in Table 2 and depicted in Figure 3C. Whereas higher values reflect better performance for asterisk task accuracy, lower values reflect better performance (i.e., faster responses) for reaction time. We assessed differences in RT across trial conditions using a 2 (trial type) × 2 (trial presentation) repeated-measures ANOVA and report the full results in Table 3. There was a significant main effect of trial type, which reflected faster RTs during control compared to experimental trials. Additionally, there was a main effect of trial presentation, with faster RTs during blocked compared to alternating trials. Finally, and critically, there was an interaction between trial type and trial presentation that was driven by a significant difference in RTs during control trials (blocked control < alternating control; *M_diff_* = 40 ms), *t*(135) = 5.84, *p* < .001, *d* = .50, but not between blocked and alternating experimental trials (*M_diff_* = 11 ms), *t*(135) = 1.78, *p* = .077, *d* = .15. Additionally, we conducted an unregistered paired-samples t-test comparing RT during alternating control and experimental trials, which confirmed predictions from the dual process model such that RTs were faster (i.e., less impairment) during alternating control trials relative to alternating experimental trials, *t*(135) = 5.59, *p* < .001, *d* = .48. Together, the pattern of RTs was consistent with a dual-process model.

#### Short-term Memory Accuracy

The average number of words recalled per short-term memory trial is reported in Table 2 and depicted in Figure 3D. We submitted the average number of words recalled to a 2 (trial type) × 2 (trial presentation) repeated-measures ANOVA. The full results are reported in Table 3. There was a main effect of trial presentation (blocked trials > alternating trials), but the main effect of trial type and its interaction with trial presentation were not significant. Thus, there was no evidence of a dual-process model reflected in short-term memory task accuracy.

#### Prospective Memory Accuracy

Prospective memory accuracy was measured as the proportion of PM trials in which participants made a PM response (i.e., pressed Enter). We calculated PM accuracy separately for blocked and alternating experimental trials, which is reported in Table 2. A paired-sample t-test revealed that PM accuracy was higher when the trials were blocked (*M* = .84, *SD* = .26) than when they alternated (*M* = .78, *SD* = .28), *t*(135) = 2.4, *p* = .018, *d* = .21. The dual-process model predicts equivalent PM accuracy across blocked and alternating experimental trials because strategic monitoring, which supports PM accuracy, is expected to be equivalent during these trials (i.e., both retrieval mode and target checking are expected to be active). Therefore, evidence of the dual-process model was not reflected in PM accuracy, and these findings conflict with Guynn’s (2003) findings, which showed no difference in PM accuracy across experimental trials. This result is also not supportive of alternative one-process accounts (i.e., retrieval mode only or target checking only), which similarly predict equal PM accuracy across experimental trials. Thus, the observed difference in PM accuracy cannot definitively support any single model and may instead reflect differences in other factors (e.g., sample size, task).

### Discussion

Despite several changes to Guynn’s (2003) original approach (i.e., recording independent measures of asterisk task accuracy and RT, and analyzing short-term memory task performance), Experiment 1 replicated a dual-process model of strategic monitoring. Specifically, we revealed evidence of a dual-process model in the number of asterisk task responses and asterisk task RT. This is consistent with findings from Guynn (2003), who showed a statistically supported dual-process model pattern in the number of asterisk task responses and a corresponding numerical pattern in asterisk task RT. Interestingly, however, the pattern indicative of a dual-process model did not emerge in the number of correctly recalled short-term memory words or in the proportion of correct asterisk responses. However, the lack of evidence in the number of short-term memory words recalled is not necessarily surprising given that participants generally performed very well on this task, remembering roughly 4 out of 5 words on each trial with very little variability across trial conditions. A similar explanation could apply to the lack of evidence in the proportion of correct asterisk responses: participants generally had very high accuracy, performing near ceiling, and with very little variability. Near-ceiling performance on these accuracy metrics prevents a definitive test of the dual-process model, and therefore the observed null effects cannot be interpreted as evidence against the model. Moreover, the fact that evidence of a dual-process model emerged in asterisk RT but not proportion correct is in line with many existing strategic monitoring studies, which show that PM demands often impair ongoing task RT but not accuracy (e.g., Anderson et al., 2019; Bugg & Ball, 2017; Einstein & McDaniel, 2005; Kuhlmann & Rummel, 2014; Lourenço et al., 2013; Lourenço & Maylor, 2014; Peper & Hunter Ball, 2023). Together, these results extend work from Guynn (2003), offering additional support for her dual-process model of strategic monitoring.

## Experiment 2

To the authors’ knowledge, there has been no systematic test of the extent to which the key findings from Guynn (2003) replicate in a more traditional PM paradigm that utilizes a single ongoing task. Therefore, the primary aim of Experiment 2 was to conceptually replicate evidence of a dual-process model in a paradigm that utilizes a LDT, a single ongoing task that is traditionally used within the PM literature (e.g., Einstein et al., 2005; Marsh et al., 2002, 2003).

### Predictions

If evidence of a dual-process model of strategic monitoring is replicable in a paradigm utilizing a LDT, then we predicted that impairment to LDT RT would mimic the cost pattern in Guynn (2003). Importantly, because existing research in which LDT is used as the ongoing task (e.g., Anderson et al., 2019; Bugg & Ball, 2017; Einstein & McDaniel, 2005; Kuhlmann & Rummel, 2014; Lourenço et al., 2013; Lourenço & Maylor, 2014; Peper & Hunter Ball, 2023) consistently shows strategic monitoring induces a cost to RT and not accuracy, it is possible that this effect, unlike Experiment 1, may be specific to RT and not accuracy. Although unexpected, it is possible to find evidence of a dual-process model in ongoing task accuracy, namely, a main effect of trial type (accuracy during experimental trials < accuracy during control trials) and an interaction between trial type and trial presentation that is driven by a significant difference between blocked and alternating control trials (accuracy during alternating < accuracy during blocked), but not between blocked and alternating experimental trials. This pattern would reflect a dual-process model of strategic monitoring in which one process (i.e., retrieval mode) is hard to turn on and off and is active on experimental trials and alternating control trials but not on blocked control trials. The other process (i.e., target checking) is easier to switch in and out of and is active on experimental trials, but not on control trials.

### Method

#### Participants and Design

An a priori power analysis determined that 136 participants were needed to detect an interaction between trial type and trial presentation with an effect size of η_p_^2^ = .07 (see effect size estimate based on Guynn, 2003, in the Methods of Experiment 1), an alpha level of .05, and 90% power using a repeated-measures ANOVA (Gpower, v.3.1; Franz et al., 2007). Therefore, 136 participants from Washington University in St. Louis completed this study for course credit. All participants received informed consent and all experimental procedures were approved by Washington University’s Institutional Review Board. Participant demographics are reported in Table 1.

**Figure 4:**
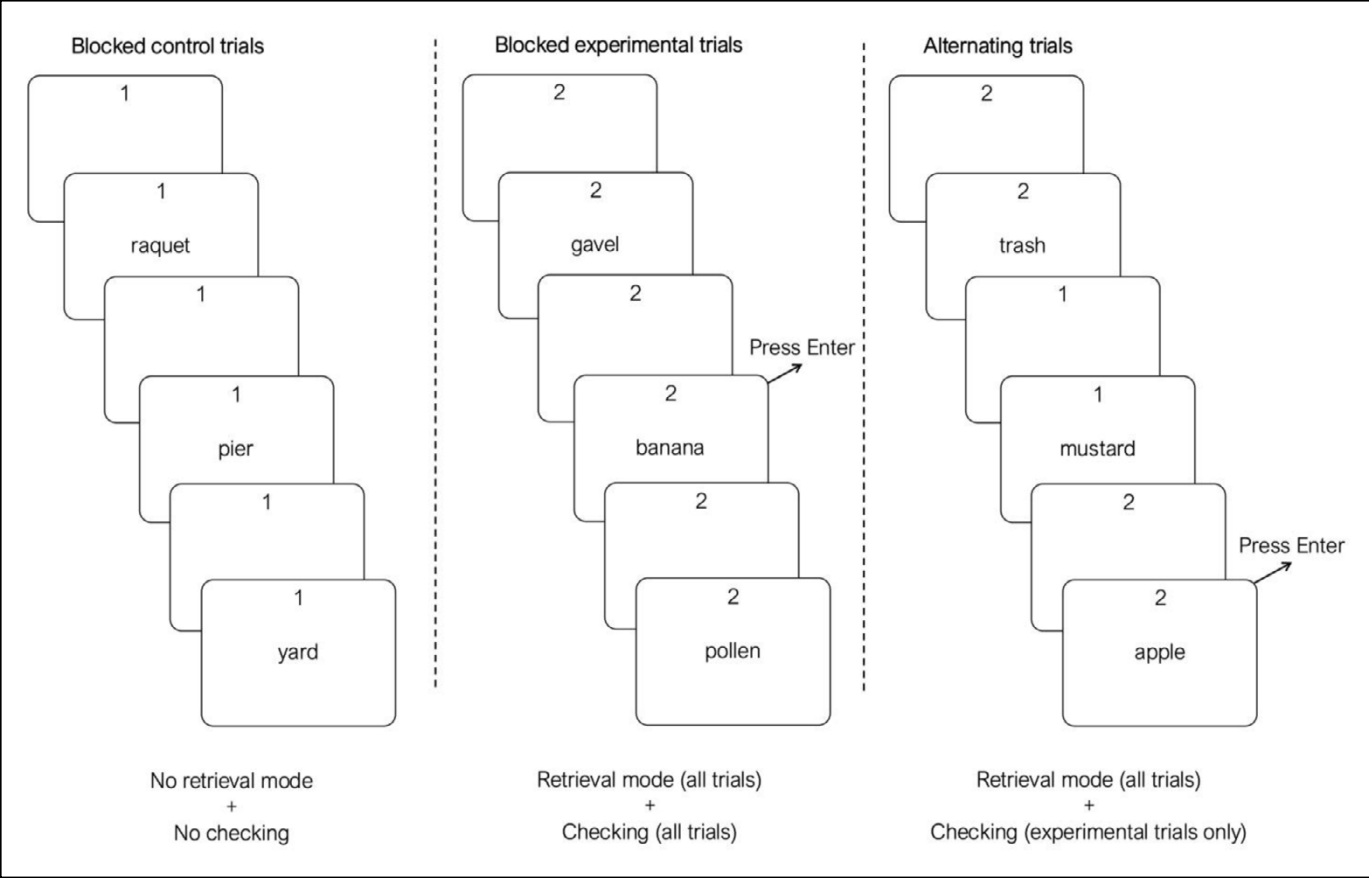
*Experiment 2 procedures*. Example blocked control trials (leftmost panel), blocked experimental trials (middle panel), and alternating blocked and experimental trials (rightmost panel). During blocked control trials, denoted with a “1” at the top of the screen, participants performed the LDT only. During blocked experimental trials, denoted with a “2” at the top of the screen, participants completed the LDT and PM tasks concurrently. During alternating trials, participants alternated between performing the LDT only (i.e., control trials) and performing the LDT with the PM task (i.e., experimental trials).

The design was a 2 (trial type: control, experimental) × 2 (trial presentation: blocked, alternating) within-subjects design. On control trials, participants performed the LDT only and on experimental trials, participants performed the LDT and PM tasks concurrently. Control and experimental trials were presented as either blocked (Figure 4, leftmost and middle panels) or alternating (Figure 4, rightmost panel). Trial type (experimental or control) and trial presentation (blocked or alternating) order was counterbalanced across participants.

#### Materials

Stimuli for the LDT were 270 of the words from Toglia and Battig (1978) semantic word norms used in Experiment 1 (Toglia & Battig, 1978). Additionally, 270 nonwords from the English Lexicon Project (Balota et al., 2007) were used. Sixty words and nonwords were randomly chosen as the stimuli for the practice trials, and the remaining 480 words and nonwords were used for test trials. We used the same practice target words (animals: dog, lion, bear, cat) and test target words (fruits: apple, peach, banana, plum, cherry, grape, apricot, pear) as Experiment 1.

#### Procedures

The experiment was administered on a computer in an individual testing room. An experimenter was in the room while participants read the instructions and completed the practice phase to answer questions and ensure participants understood the instructions.

Participants were told that they would perform two tasks, and that they would receive practice performing one task alone before practicing both tasks together. Participants first practiced the LDT alone (i.e., control trials). They were informed that strings of letters would appear in the center of the screen, and that they should decide whether the string of letters is a word or a nonword as quickly and accurately as possible. They were instructed to press the *J* key if the string of letters forms a real word, and the *F* key if the string of letters is not a real word. Participants were also told that a “1” would appear at the top of the screen before these trials to indicate that they should only perform the LDT during these trials. The “1” appeared for 1 second before the start of each control trial and remained there for the duration of the trial. Participants received feedback on the accuracy and speed of their response after each key press. Participants completed 12 self-paced practice LDT trials, with a 500 millisecond intertrial interval before the next “1” appeared at the top of the screen indicating the start of a new control trial.

Next, participants were told that they would complete a second task in addition to the LDT (i.e., experimental trials). Participants were instructed that they would continue to make word and nonword judgements as quickly and as accurately as possible, but that they should make a special response (press *Enter*) if an animal word appears on the screen during the LDT. They were informed that a “2” would appear at the top of the screen before these trials, indicating that they should perform the LDT and PM tasks together. The “2” appeared 1 second before the start of each experimental trial and remained there for the duration of the trial. Participants completed 48 practice experimental trials during which they received feedback on the accuracy and speed of their responses. A target word appeared on trials 8, 20, 32, and 44. If a participant failed to detect a target word, they received feedback reminding them of the PM task. Each trial was followed by a 500 millisecond intertrial interval before a “2” appeared at the top of the screen indicating the start of a new experimental trial.

At the end of the practice phase, participants were informed that they would move on to the test trials. They were instructed that, as practiced, some trials would require them to perform just the LDT and these trials were indicated with a “1” at the top of the screen. Other trials required them to perform the LDT and PM tasks together, and these trials were denoted with a “2”. Participants were informed that they would not receive feedback on their performance during test trials. Additionally, participants were told about whether the upcoming block would be blocked or alternating prior to the start of each block.

Test trials were organized into 8 blocks of 60 trials for a total of 480 trials. There was a 10 second rest between blocks. Consistent with Experiment 1, the order of trial presentation (blocked vs. control) was counterbalanced across participants. For half the participants, the first 240 trials consisted of 120 blocked control trials and 120 blocked experimental trials, followed by 240 alternating control and experimental trials. The other half of the participants completed 240 alternating control and experimental trials followed by 120 blocked control trials and 120 blocked experimental trials. Additionally, trial type (control vs. experimental) order was counterbalanced across participants, with half the participants starting with experimental trials and half starting with control trials. The first trial of alternating and blocked trials was the same trial type. In other words, if a participant started with a blocked control trial, they also began with an alternating control trial.

The number of test trials in Experiment 2 was larger than in Experiment 1 and in Guynn (2003), which consisted of 96 short-term memory test trials. However, participants in Experiment 1 and Guynn (2003) made multiple asterisk responses per short-term memory trial. Based on the numbers reported in Guynn (2003), participants made an average of five asterisk responses per short term memory trial for a total of roughly 480 observations from which to measure ongoing task accuracy and RT. To match this aspect of the original design and to ensure we have enough observations to detect a cost pattern, Experiment 2 included 480 test trials.

#### Statistical Analyses

All statistical analysis were completed in R Statistical Software (v.4.2.2, R Core Team, 2023). Following the procedures in Guynn (2003) and Experiment 1, target trials and trials with an RT less than 100 ms were excluded from accuracy and RT analysis. Because our main aim is to test the replicability of Guynn’s (2003) key findings in a traditional ongoing task, we used the trimming procedures typically used to analyze LDT RT data in PM paradigms (e.g., Ball & Bugg, 2018a, 2018b; Bugg & Ball, 2017; Kuhlmann & Rummel, 2014; Lourenço et al., 2013; Lourenço & Maylor, 2014). Specifically, RT analyses were performed on correct trials and were trimmed at 2.5 standard deviations within each participant’s mean RT, separately for blocked and alternating experimental and control trials. Statistical tests were conducted using alpha = 0.05, and effect sizes for t-tests (Cohen’s d) and ANOVAS (partial eta squared; η_p_^2^) were reported.

##### LDT Performance

###### Accuracy

To calculate ongoing task accuracy, we calculated the proportion of correct responses during the LDT. We compared LDT accuracy using a 2 (trial type: control, experimental) × 2 (trial presentation: blocked, alternating) repeated measures ANOVA. Unlike Experiment 1, we did not necessarily expect there to be a cost to ongoing task accuracy, given that prior research using LDT as the ongoing task does not typically find a cost to accuracy associated with PM monitoring. However, if the patterns of cost to LDT accuracy mirror the pattern predicted by the dual-process model (i.e., a significant main effect of trial type and trial presentation, and a significant interaction driven by a significant difference between blocked and alternating control but not experimental trials), this would still be consistent with a model in which strategic monitoring is supported by two distinct processes.

###### Reaction time

To examine ongoing task RT, we submitted mean trimmed RTs to a 2 (trial type) × 2 (trial presentation) repeated measures ANOVA. If the cost pattern predicted by the dual-process model is replicated in a LDT, then we expect there to be significant main effects of trial type and trial presentation, indicating that people strategically monitor for PM targets during trials where PM targets are expected to occur. More importantly, we expect a significant interaction between trial type and trial presentation, and critically, this interaction should result from a significant difference in RT between blocked and alternating control trials (RT during alternating > RT during blocked) but not between blocked and alternating experimental trials. A significant interaction consistent with this pattern would evidence that strategic monitoring is a dual-process supported by retrieval mode and target checking.

##### PM Accuracy

We measured PM accuracy as the proportion of successful PM target detections during experimental trials. To test whether PM accuracy differs depending on trial presentation, we ran a paired-samples t-test comparing PM accuracy during blocked experimental and alternating experimental trials. Like Experiment 1, we did not expect PM accuracy to differ depending on whether the experimental trials were blocked or alternating.

### Results

#### LDT Performance

Less than .01% of trials were excluded from accuracy and reaction time analyses for having reaction times < 100 ms. For the reaction time analysis specifically, approximately 2.8% of trials were trimmed according to the trimming procedures detailed in the Method.

##### LDT Accuracy

**Figure 5:**
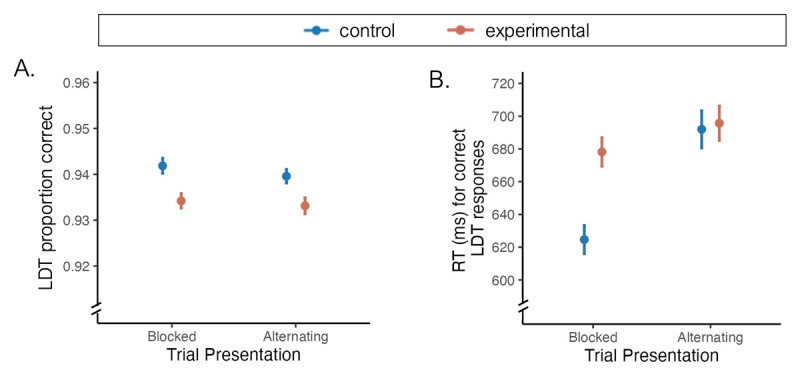
Ongoing LDT performance during Experiment 2. **A.** Proportion of correct LDT trials during each trial type and trial presentation. Larger numbers represent better performance. **B.** Mean reaction time for correct LDT responses. Lower numbers represent better performance. All error bars represent within-subject standard errors.

Ongoing LDT accuracy was measured as the proportion of correct LDT responses, and was high overall (*M* = .94, *SD* = .05). Mean LDT accuracy is reported in Table 2 and depicted in Figure 5A, separately for each trial type and trial presentation. Mean accuracy was submitted to a 2 (trial type) × 2 (trial presentation) repeated-measures ANOVA and the full results are reported in Table 3. There was a main effect of trial type, driven by higher accuracy during control relative to experimental trials. The main effect of trial presentation and its interaction with trial type were not significant.

##### LDT Reaction Time

Mean LDT RTs for correct trials are reported in Table 2 and depicted in Figure 5B. As a reminder, lower reaction times (i.e., faster responses) correspond to better performance. We submitted mean RT to a 2 (trial type) × 2 (trial presentation) repeated measures ANOVA and report the full results in Table 3. There was a main effect of trial type, driven by faster RT for control trials relative to experimental trials. The main effect of trial presentation was also significant, driven by faster RT for blocked trials relative to alternating trials. Finally, there was an interaction between trial type and trial presentation. Separate preregistered paired-sample t-tests revealed that, consistent with predictions from the dual process model, there was a significant difference between RT on blocked and alternating control trials (RT blocked < RT alternating, *M_diff_* = 67 ms), *t*(135) = 6.67, *p* < .001, *d* = .57. However, contrary to predictions from a dual process model, there was also a difference between blocked and alternating experimental trials (RT blocked < RT alternating, *M_diff_* = 18 ms), *t*(135) = 2.33, *p* = .021, *d* = .20. Next, as in Experiment 1, we conducted an unregistered paired-sample t-test to test whether RTs were faster (i.e., better performance) during alternating control relative to alternating experiment trials, as predicted by the dual-process model. Contrary to the expectations from the dual-process model, there was no difference in RT during alternating control and experimental trials, *t*(135) = 1.41, *p* = .160, *d* = .12. Collectively, the RT pattern does not support a dual-process model.

If this pattern does not support a dual-process model, could it instead reflect an alternative one-process model (i.e., retrieval mode only or target checking only)? According to a model whereby strategic monitoring is governed by retrieval mode only, participants should be in retrieval mode during all trials except blocked control trials, and therefore RTs should be fastest during blocked control trials and slower, but equivalent, in alternating control trials and all experimental trials. Although RTs were fastest during blocked control trials, they were not equivalent in all other trials because blocked experimental trials were significantly faster than alternating experimental trials and numerically faster than alternating control trials. Thus, the RT pattern is not consistent with a retrieval mode only model. Similarly, the RT pattern is not consistent with a target checking only model, which predicts that RT should be equally fast during control trials, where participants are not expected to check for targets, and equally slow during experimental trials, where participants should be target checking. Instead, RTs differed between blocked and alternating control trials, and between blocked and alternating experimental trials. Thus, the RT results do not provide evidence in support of a dual-process model, nor an alternative one-process model.

#### PM Accuracy

PM accuracy was measured as the proportion of PM trials during which participants made a PM response (Table 2). Overall mean PM accuracy was .60 (*SD* = .28) and did not differ between alternating trials (*M* = .60, *SD* = .32) and blocked trials (*M* = .61, *SD* = .31), *t*(135) = .35, *p* = .727, *d* = .03. This is consistent with predictions from the dual-process model.

### Discussion

Experiment 2 aimed to determine whether the dual-process model of Guynn (2003) would be evidenced in a more traditional ongoing task – the LDT. Although there was no evidence of a dual process model in LDT accuracy, this finding was not entirely unexpected given that existing strategic monitoring studies using an LDT as the ongoing task generally find no systematic impairment to ongoing task accuracy (i.e., proportion correct) as a result of strategic monitoring (e.g., Anderson et al., 2019; Bugg & Ball, 2017; Einstein & McDaniel, 2005; Kuhlmann & Rummel, 2014; Lourenço et al., 2013; Lourenço & Maylor, 2014; Peper & Hunter Ball, 2023). The pattern of LDT RT was less straightforward. According to the dual-process model, there should have been a difference in RT during blocked and alternating control trials, reflective of participants being in retrieval mode during alternating but not blocked control trials; in contrast, the model predicted no difference between blocked and alternating experimental trials because participants should be in retrieval mode and target checking during all experimental trials. Contrary to the latter prediction, we found a significant difference between blocked and alternating control *and* experimental trials. However, the difference between control trials (M_diff_ = 67 ms) was considerably larger than the difference between experimental trials (M_diff_ = 18 ms). Thus, although the dual-process model predicted a difference in control trials only, the fact that the mean difference was smaller for experimental compared to control trials may suggest that the two processes were operating, but evidence of such was weakened by the trial presentation manipulation, which may exert a particularly strong effect in an LDT (relative to the asterisk task in Experiment 1 and Guynn, 2003). We will discuss this possibility further in the General Discussion.

## General Discussion

The dual-process model (Guynn, 2003) has been a predominant mechanistic model of strategic monitoring since its inception over two decades ago, often being used to motivate research or explain findings. Despite its prominence, there have been no replications of the seminal findings, nor a test of whether it also characterizes monitoring processes in alternative, more traditional ongoing tasks, such as the LDT. Thus, this study aimed to provide a conceptual replication (Experiment 1) and extension (Experiment 2) of Guynn’s (2003) findings to test the robustness and generalizability of the dual-process model of strategic monitoring. Experiment 1 largely replicated findings from Guynn (2003) using a combination of asterisk and short-term memory tasks as the ongoing tasks, providing the first conceptual replication of the dual-process model. In contrast, using a single, and more traditional ongoing task, LDT, Experiment 2 provided little support for the dual-process model suggesting the two processes may operate differently depending on the nature of the ongoing task(s). Next, we will discuss the findings and implications of each experiment in more depth.

### Summary of Findings Testing the Dual-Process Model

#### Ongoing task performance

Despite several changes to the paradigm to allow for a more comprehensive test of the theory, Experiment 1 provided support for a dual-process model. Specifically, evidence emerged in the same two ongoing task performance metrics used in Guynn (2003): the number of correct asterisk responses and asterisk task RT. Notably, whereas Guynn found numerical but not statistical support of the dual-process model in asterisk task RT, Experiment 1 provided statistical support of the model in this measure. This difference likely reflects our modification to the original paradigm, which disentangled asterisk task RT from accuracy (i.e., proportion correct), two measures that were confounded in the original design. Results from two additional performance metrics – asterisk task proportion correct and the number of correctly recalled short-term memory words, which were reported in the present study but not Guynn– revealed no evidence of a dual-process model. However, as noted earlier, participants were generally very accurate when responding to the asterisk and short-term memory tasks, performing nearly at ceiling during both tasks and with little individual variability. This near-ceiling performance limits the ability to definitively test the dual-process model, and therefore the observed null effects cannot be construed as evidence for or against the model. In addition, the results from Experiment 1 are concordant with the majority of existing strategic monitoring studies (e.g., Anderson et al., 2019; Bugg & Ball, 2017; Einstein & McDaniel, 2005; Kuhlmann & Rummel, 2014; Lourenço et al., 2013; Lourenço & Maylor, 2014; Peper & Hunter Ball, 2023), which reliably show strategic monitoring effects in RT but not accuracy. In sum, Experiment 1 provided the first conceptual replication of Guynn’s findings, thereby lending further support to the dual-process model.

The findings from Experiment 2, using a similar design as Experiment 1 except with a more traditional PM paradigm that uses a single ongoing task (LDT), were less straightforward. As in Experiment 1 and consistent with the broader strategic monitoring literature, there was no evidence of a dual process model in LDT accuracy (i.e., proportion correct). Participants once again demonstrated near-ceiling accuracy with little variability, rendering the results uninterpretable. More surprising, however, was the pattern that emerged in LDT RT, which is where evidence of a dual-process model was expected to appear. Consistent with a central prediction of the dual-process model, there was a significant interaction between trial type and trial presentation; however, the specific pattern of results deviated somewhat from model expectations. Instead of the model’s predicted pattern – a difference between blocked and alternating control trials but not experimental trials – we found that RTs were faster (i.e., better performance) for blocked control *and* experimental trials compared to their alternating counterparts. Moreover, whereas the model assumes better performance during alternating control compared to alternating experimental trials, reflecting engagement of target checking during alternating experimental but not control trials, we found no difference in RTs between these trial types. Thus, Experiment 2 provides no clear evidence of a dual-process model. Moreover, the pattern does not align with predictions from alternative one-process models. At first glance, the pattern appears akin to predictions from a retrieval-mode only model: high performance during blocked control trials (participants are not in retrieval mode), and low but equal performance during all other trials (participants are in retrieval mode). Although similar, the pattern of results from Experiment 2 is not consistent with this model because performance during blocked experimental trials and alternating control and experimental trials was not equivalent. We will consider potential alternative explanations for these findings and implications for future investigations in subsequent sections.

#### PM Accuracy

The dual-process model predicts that PM accuracy should be equivalent in blocked and alternating experimental trials because the processes operating during these trials should be the same (i.e., retrieval mode and target checking). Consistent with this, PM accuracy during Experiment 2 was equivalent in blocked and alternating experimental trials. However, PM accuracy during Experiment 1 was higher during blocked experimental trials than during alternating experimental trials. One potential explanation for this finding is that the same processes were operating during blocked and alternating experimental trials, allowing for comparable PM target detection, but that the alternating trials imposed greater attentional demands (e.g., needing to check the “2” or “3” trial cue to know when to check for targets; turning on and off checking) that impaired PM performance. In fact, evidence from Guynn (2003) suggests a similar trend. Specifically, Guynn reported numerically higher PM accuracy during blocked trials (*M* = .84) than during alternating trials (*M* = .79), but this difference was not significant. Notably, these means closely parallel those observed in Experiment 1 (blocked *M* = .84; experimental *M* = .78). Therefore, it is plausible that the larger sample size in the present study provided sufficient statistical power to detect an effect that went undetected in Guynn (2003). Taken together with the evidence in support of a dual-process model from ongoing task performance, it seems likely that the difference in PM accuracy during Experiment 1 is a result of differences in attentional demands across experimental trials rather than differences in the underlying processes.

### Potential Explanations for the Lack of Evidence in Support of Dual-Process Model in LDT

#### Differences in Task Timing

Why might evidence of a dual-process model emerge when indexed in the ongoing tasks used in Guynn (2003) and Experiment 1, but not in an LDT? One potential explanation is that the dual-process model is operating during Experiment 2, but its footprint is obscured by the influence of task characteristics that are specific to the LDT (compared to the asterisk task in Experiment 1, which yielded evidence for the dual-process model). For example, one key difference in ongoing task characteristics across experiments is the duration of each trial. In Experiment 1 (and Guynn, 2003), each trial lasted a fixed amount of time: 11.5 seconds (1.5 second trial cue + 9 second to perform the ongoing task). In Experiment 2, however, each trial was much shorter. Even trials with the slowest RTs lasted less than 3 seconds (1.5 second trial cue + 1.4 seconds performing the ongoing task), which is nearly 4 times shorter than trials during Experiment 1. The difference in trial timing may be particularly important when considering the demands during alternating trials. Participants in Experiment 1 alternated trial types every 11.5 seconds whereas participants in Experiment 2 alternated trial types every 2–3 seconds. The more rapid alternations in Experiment 2 (compared to Experiment 1 and Guynn, 2003) may have limited participants’ willingness or ability to flexibly engage and disengage target checking processes during alternating experimental and control trials. Prior research shows that adjusting internal attentional states may impose a cost (Grahek et al., 2023), which deters people from expending effort to switch between such states (Ileri-Tayar et al., 2024). This raises the possibility that turning on and turning off target checking could similarly be costly and thus might be avoided when it must be done quite frequently as in Experiment 2. Said differently, given the short duration and rapid alternation of trials, it may have been less demanding for participants to simply check on all trials rather than turning it off every other trial, and this aligns with the finding showing equal costs for both trial types (control and experimental).

If the rapid alternation between trial types imposes a cost, as described above, this could also help explain the unexpected difference between blocked and alternating experimental trials. Specifically, the rapid trial type alternations may increase attentional demands, leading to an additional impairment to ongoing task performance during alternating experimental trials relative to blocked experimental trials. Thus, the difference between the blocked and alternating experimental trials could reflect differences in attentional demands rather than a difference in the underlying processes across experimental trials. One piece of evidence supporting this interpretation is that the difference between blocked and alternating control trials (*M*_diff_ = 67 ms) was considerably larger than that for experimental trials (*M*_diff_ = 18 ms). This pattern indicates that, beyond a general increase in task demands during alternating trials, additional factors – presumably differences in the underlying processes engaged during experimental trials and alternating control trials, consistent with the dual-process model – contributed to the observed pattern.

If the pattern observed in Experiment 2 was indeed driven by a decreased willingness to disengage target checking when the trial types alternate frequently, this does not necessarily rule out the existence of a dual-process model. Rather, it illustrates a boundary condition for its observation, revealing how task characteristics can influence when and how individuals engage in retrieval mode and target checking. Specifically, we may have inadvertently identified a condition in which the engagement and disengagement of these processes becomes less efficient, namely when the trial type alternations become more rapid (i.e., fast). This finding has important implications for interpreting prior research, underscoring the role of task structure on engagement of strategic monitoring processes. For example, it can help explain findings from existing PM studies (for a review and meta-analysis, see Peper & Hunter Ball, 2023), which show a stronger strategic monitoring effect when the context (analogous to trial type in the present study) switches block-by-block (i.e., infrequently) compared to trial-by-trial (i.e., frequently). In these cases, the weaker strategic monitoring patterns in the trial-by-trial design may specifically reflect difficulty disengaging target checking during irrelevant PM contexts (i.e., where PM targets do not occur; control trials), a limitation imposed by rapid context switches. More broadly, findings from Experiment 2 emphasize the importance of considering task characteristics when designing future studies, particularly how they influence when and to what extent retrieval mode and target checking operate to support strategic monitoring.

#### Differences in Number of Ongoing Tasks

A second potential explanation for why we observed evidence of a dual-process model in Experiment 1 but not Experiment 2 is that the experiments involved a different number of ongoing tasks. In Experiment 1, participants performed two ongoing tasks, the asterisk and short-term memory tasks, whereas participants in Experiment 2 performed only the LDT. The additional ongoing task in Experiment 1 likely increased the amount of attentional resources required to perform the ongoing tasks, making strategic allocation of the limited remaining resources more critical. In other words, the constrained attentional resources may have required participants to be more efficient in their engagement and disengagement of retrieval mode and target checking such that they only allocated resources when necessary. In contrast, participants in Experiment 2 may not have experienced the same limitations on their attentional resources given that they were only performing one ongoing task. As a result, they may not have needed to allocate attention to retrieval mode and target checking as strategically or efficiently. If so, we might expect LDT performance to be the same in all trials because participants had sufficient attentional resources to stay in retrieval mode and check for targets on all trials. However, this was not the pattern we observed. Thus, the number of ongoing tasks alone does not appear to explain the differing results across the two experiments.

## Conclusion

In sum, this study provided the first conceptual replication of Guynn’s (2003) findings providing further support for the dual-process model of strategic monitoring, which posits that strategic monitoring is supported by both retrieval mode and target checking. Moreover, this was the first study to test the replicability of the evidence supporting the dual-process model in a traditional and commonly used ongoing task: the LDT. Notably, there was no clear evidence of a dual-process model in the LDT, suggesting that engagement of retrieval mode and target checking processes may be susceptible to the influence of task characteristics such as the timing of each trial. Together, this study advances our understanding of the mechanisms underlying strategic monitoring in PM and provides important insights into how task demands shape the footprint of the dual-process model of strategic monitoring.

## Data Availability

Data and analysis code for this study are publicly available on Open Science Framework (https://osf.io/9vrzu).

## Footnotes

Guynn (2003) did not report *p*-values or η_p_^2^, however, we computed these statistics and include them here. We calculated p-values using degrees of freedom and *F*-statistics. To calculate η_p_^2^ for each effect, we used MSE, F-statistics, and degrees of freedom to compute the sum of squares for the effect and for the error, which were used to calculate partial eta-squared.

## References

[1] Anderson, F. T., Strube, M. J., & McDaniel, M. A. (2019). Toward a better understanding of costs in prospective memory: A meta-analytic review. Psychological Bulletin, 145(11), 1053–1081. 10.1037/bul0000208 31464456

[2] Ball, B. H., & Bugg, J. M. (2018a). Aging and the strategic use of context to control prospective memory monitoring. Psychology and Aging, 33(3), 527–544. 10.1037/pag0000247 29756806 PMC5954997

[3] Ball, B. H., & Bugg, J. M. (2018b). Context cue focality influences strategic prospective memory monitoring. Psychonomic Bulletin & Review, 25(4), 1405–1415. 10.3758/s13423-018-1442-9 29435962 PMC6070398

[4] Ball, B. H., Li, Y. P., & Bugg, J. M. (2020). Aging and strategic prospective memory monitoring. Memory & Cognition, 48(3), 370–389. 10.3758/s13421-019-00976-8 31628616 PMC11258997

[5] Ball, B. H., Peper, P., & Bugg, J. M. (2024). When is context used to guide prospective memory monitoring? Psychonomic Bulletin & Review. 10.3758/s13423-024-02568-3 39313676

[6] Balota, D. A., Yap, M. J., Hutchison, K. A., Cortese, M. J., Kessler, B., Loftis, B., Neely, J. H., Nelson, D. L., Simpson, G. B., & Treiman, R. (2007). The English Lexicon Project. Behavior Research Methods, 39(3), 445–459. 10.3758/BF03193014 17958156

[7] Bowden, V. K., Smith, R. E., & Loft, S. (2021). Improving prospective memory with contextual cueing. Memory & Cognition, 49(4), 692–711. 10.3758/s13421-020-01122-5 33420709

[8] Bugg, J. M. (2017). Context, Conflict, and Control. In T. Egner (Ed.), The Wiley Handbook of Cognitive Control (1st ed., pp. 79–96). Wiley. 10.1002/9781118920497.ch5

[9] Bugg, J. M., & Ball, B. H. (2017). The strategic control of prospective memory monitoring in response to complex and probabilistic contextual cues. Memory & Cognition, 45(5), 755–775. 10.3758/s13421-017-0696-1 28275948

[10] Cohen, A.-L., Jaudas, A., Hirschhorn, E., Sobin, Y., & Gollwitzer, P. M. (2012). The specificity of prospective memory costs. Memory, 20(8), 848–864. 10.1080/09658211.2012.710637 22900905

[11] Einstein, G. O., Holland, L. I., Guynn, M. J., & McDaniel, M. A. (1992). Age-Related Deficits in Prospective Memory: The Influence of Task Complexity. Psychology and Aging, 7(3), 471–478. https://doi-org.ccl.idm.oclc.org/10.1037/0882-7974.7.3.471 1388869 10.1037//0882-7974.7.3.471

[12] Einstein, G. O., & McDaniel, M. A. (1990). Normal aging and prospective memory. Journal of Experimental Psychology: Learning, Memory, and Cognition, 16(4), 717–726. 10.1037/0278-7393.16.4.717 2142956

[13] Einstein, G. O., & McDaniel, M. A. (2005). Prospective Memory: Multiple Retrieval Processes. Current Directions in Psychological Science, 14(6), 286–290. 10.1111/j.0963-7214.2005.00382.x

[14] Einstein, G. O., McDaniel, M. A., Thomas, R., Mayfield, S., Shank, H., Morrisette, N., & Breneiser, J. (2005). Multiple Processes in Prospective Memory Retrieval: Factors Determining Monitoring Versus Spontaneous Retrieval. Journal of Experimental Psychology: General, 134(3), 327–342. 10.1037/0096-3445.134.3.327 16131267

[15] Franz, F., Erdfelder, E., Lang, A.-G., & Buchner, A. (2007). G*Power 3: A flexible statistical power analysis program for the social, behavioral, and biomedical sciences. Behavior Research Methods, 39(2), 175–191. 10.3758/BF03193146 17695343

[16] Grahek, I., Leng, X., Musslick, S., & Shenhav, A. (2023). Control adjustment costs limit goal flexibility: Empirical evidence and a computational account. Neuroscience. 10.1101/2023.08.22.554296 PMC13023295 40720280

[17] Guynn, M. J. (2003). A two-process model of strategic monitoring in event-based prospective memory: Activation/retrieval mode and checking. International Journal of Psychology, 38(4), 245–256. 10.1080/00207590344000178

[18] Ileri-Tayar, M., Colvett, J. S., & Bugg, J. M. (2024). Between-task transfer of item-specific control is replicable and extends to novel conditions. Journal of Experimental Psychology: Human Perception and Performance, 50(6), 535–553. 10.1037/xhp0001200 38573694

[19] Kuhlmann, B. G., & Rummel, J. (2014). Context-specific prospective-memory processing: Evidence for flexible attention allocation adjustments after intention encoding. Memory & Cognition, 42(6), 943–949. 10.3758/s13421-014-0405-2 24590428

[20] Lourenço, J. S., & Maylor, E. A. (2014). Is it Relevant? Influence of Trial Manipulations of Prospective Memory Context on Task Interference. Quarterly Journal of Experimental Psychology, 67(4), 687–702. 10.1080/17470218.2013.826257 23971415

[21] Lourenço, J. S., White, K., & Maylor, E. A. (2013). Target context specification can reduce costs in nonfocal prospective memory. Journal of Experimental Psychology: Learning, Memory, and Cognition, 39(6), 1757–1764. 10.1037/a0033702 23834056

[22] Marsh, R. L., Hicks, J. L., & Cook, G. I. (2006). Task interference from prospective memories covaries with contextual associations of fulfilling them. Memory & Cognition, 34(5), 1037–1045. 10.3758/BF03193250 17128602

[23] Marsh, R. L., Hicks, J. L., Cook, G. I., Hansen, J. S., & Pallos, A. L. (2003). Interference to Ongoing Activities Covaries With the Characteristics of an Event-Based Intention. Journal of Experimental Psychology. Learning, Memory, and Cognition, 29(5), 861–870. 10.1037/0278-7393.29.5.861 14516219

[24] Marsh, R. L., Hicks, J. L., & Watson, V. (2002). The dynamics of intention retrieval and coordination of action in event-based prospective memory. Journal of Experimental Psychology: Learning, Memory, and Cognition, 28(4), 652–659. 10.1037/0278-7393.28.4.652 12109759

[25] McDaniel, M. A., Guynn, M. J., Einstein, G. O., & Breneiser, J. (2004). Cue-Focused and Reflexive-Associative Processes in Prospective Memory Retrieval. Journal of Experimental Psychology: Learning, Memory, and Cognition, 30(3), 605–614. 10.1037/0278-7393.30.3.605 15099129

[26] Peper, P., & Hunter Ball, B. (2023). Strategic monitoring improves prospective memory: A meta-analysis. Quarterly Journal of Experimental Psychology, 76(11), 2546–2569. 10.1177/17470218231161015 PMC10585947 37010132

[27] R Core Team. (2023). R: A Language and Environment for Statistical Computing (Version 4.3.2) [Computer software]. R Foundation for Statistical Computing. https://www.R-project.org/

[28] Scullin, M. K., McDaniel, M. A., & Shelton, J. T. (2013). The Dynamic Multiprocess Framework: Evidence from prospective memory with contextual variability. Cognitive Psychology, 67(1–2), 55–71. 10.1016/j.cogpsych.2013.07.001 23916951 PMC3809757

[29] Smith, R. E. (2003). The cost of remembering to remember in event-based prospective memory: Investigating the capacity demands of delayed intention performance. Journal of Experimental Psychology: Learning, Memory, and Cognition, 29(3), 347–361. 10.1037/0278-7393.29.3.347 12776746

[30] Smith, R. E., & Bayen, U. J. (2006). The source of adult age differences in event-based prospective memory: A multinomial modeling approach. Journal of Experimental Psychology: Learning, Memory, and Cognition, 32(3), 623–635. 10.1037/0278-7393.32.3.623 16719671

[31] Smith, R. E., Hunt, R. R., & Murray, A. E. (2017). Prospective memory in context: Moving through a familiar space. Journal of Experimental Psychology: Learning, Memory, and Cognition, 43(2), 189–204. 10.1037/xlm0000303 27504679 PMC5290035

[32] Toglia, M. P., & Battig, W. F. (1978). Handbook of semantic word norms. Lawrence Erlbaum Associates, Inc.

